# HGF/c-MET: A Promising Therapeutic Target in the Digestive System Cancers

**DOI:** 10.3390/ijms19113295

**Published:** 2018-10-23

**Authors:** Hongli Zhang, Qingqing Feng, Wei-Dong Chen, Yan-Dong Wang

**Affiliations:** 1 State Key Laboratory of Chemical Resource Engineering, College of Life Science and Technology, Beijing University of Chemical Technology, Beijing 100029, China; hlz13084@163.com (H.Z.); fengqing910524@163.com (Q.F.); 2 Key Laboratory of Molecular Pathology, School of basic medical science, Inner Mongolia Medical University, Hohhot 010110, China; 3 Key Laboratory of Receptors-Mediated Gene Regulation and Drug Discovery, School of Medicine, Henan University, Kaifeng 475004, China

**Keywords:** HGF, c-MET, digestive system cancers, miRNA

## Abstract

The HGF/c-MET pathway is active in the development of digestive system cancers, indicating that inhibition of HGF/c-MET signaling may have therapeutic potential. Various HGF/c-MET signaling inhibitors, mainly c-MET inhibitors, have been tested in clinical trials. The observed efficacy and adverse events of some c-MET inhibitors were not very suitable for treating digestive system cancers. The development of new HGF/c-MET inhibitors in preclinical studies may bring promising treatments and synergistic combination (traditional anticancer drugs and c-MET inhibitors) strategies provided anacceptable safety and tolerability. Insights into miRNA biology and miRNA therapeutics have made miRNAs attractive tools to inhibit HGF/c-MET signaling. Recent reports show that several microRNAs participate in inhibiting HGF/c-MET signaling networks through antagonizing c-MET or HGF in digestive system cancers, and the miRNAs-HGF/c-MET axis plays crucial and novel roles for cancer treatment. In the current review, we will discuss recent findings about inhibitors of HGF/c-MET signaling in treating digestive system cancers, and how miRNAs regulate digestive system cancers via mediating HGF/c-MET pathway.

## 1. Introduction

Digestive tract malignancies, mainly including gastric cancer, hepatocellular carcinoma (HCC), pancreatic cancer, esophageal cancer, and colon and rectal cancer, are highly common cancers and causes of cancer deaths worldwide [1]. Despite surgery, chemotherapy and molecular targeted therapy have been widely used in cancer treatment, median survival for most stage IV digestive cancers is <1 year, with the exception of small bowel and colorectal adenocarcinoma [2]. Thus, it was essential and imperative to explore and identify novel and effective therapeutic targets for the prevention and treatment of digestive system cancers.

**Figure 1:**
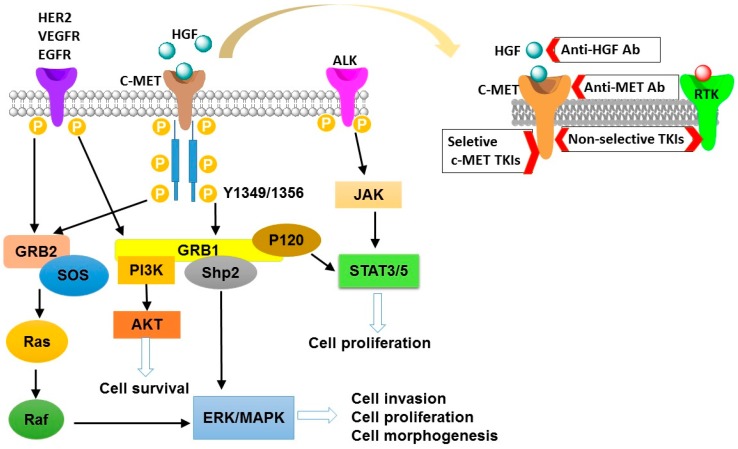
The schematic diagram of c-mesenchymal–epithelial transition (c-MET) activation signaling pathways. Activated c-MET binds adaptor molecules GRB-1 and GRB-2, and then they recruit Ras/Raf, SH2 containing protein tyrosine phosphatase (Shp2), STAT3/5, and PI3K signals. In addition, activation of other receptor tyrosine kinases (RTKs) signaling molecules (HER2, VEGFR, EGFR, and ALK) may cross-talk with the c-MET, further recruiting PI3K, ERK, and STAT3/5 signals. These downstream signals are involved in regulating cell morphogenesis, survival, proliferation, and invasion. Inhibition of the HGF/c-MET pathway can be achieved through c-MET kinase inhibitors (TKIs), anti-HGF monoclonal antibodies (Anti-HGF Ab), and anti-MET monoclonal antibodies (Anti-MET Ab). HGF, hepatocyte growth factor; ALK, anaplastic lymphoma kinase; JAK, Janus kinase; STAT3/5, signal transducers and activators of transcription 3/signal transducers and activators of transcription 5; HER2, human epidermal growth factor receptor 2; VEGFR, vascular endothelial growth factor receptor 2; EGFR, epidermal growth factor receptor; P, phosphorylation; GRB, growth factor receptor-bound protein; SOS, son of sevenless; Ras, GTPases and key transducers of receptor tyrosine kinase; Raf, raf kinase, effector of Ras; PI3K, phosphatidylinositide 3-kinases; AKT, protein kinase B; ERK, extracellular regulated protein kinases; MAPK, mitogen-activated protein kinase.

The c-mesenchymal–epithelial transition (c-MET), a kinase receptor for hepatocyte growth factor (HGF), is well-known for its roles in driving tumorigenesis [3,4,5]. It is a disulfide-linked heterodimer consisting of a highly glycosylated extracellular α-subunit and a transmembrane β-subunit. Upon binding to the HGF, c-MET triggers dimerization of two subunits, leading to autophosphorylation of tyrosine residues in cytoplasmic domain [4,6,7]. Then, phosphorylation of these tyrosine residues (Tyr^1349^ and Tyr^1356^) results in an activated C-terminal docking site, which has been identified to be able to recruit intracellular adaptor proteins [8]. These events trigger several downstream signaling pathways such as phosphoinositide 3-kinase/threonine-protein kinase (PI3K/AKT) pathway, wingless-related integration site (Wnt) pathway, and others [6,9,10]. Moreover, HGF/c-MET induced cell proliferation, migration, survival, invasion, differentiation, and epithelial–mesenchymal transition (EMT), promoting the progression of tumorigenesis [4,11,12]. These common downstream signaling pathways for HGF/c-MET activation has summarized in Figure 1.

The HGF/c-MET receptor tyrosine kinase (RTK) pathway is quiescent in normal tissue, while it is active in various tumors [13]. Growing investigations have confirmed that inhibition of HGF/c-MET signaling is an effective therapeutic strategy in suppressing multiple human cancers, such as non-small cell lung cancer (NSCLC), HCC, gastric cancer, colorectal cancer ovarian cancer, bladder cancer, head and neck cancer, cervical cancer, and some other cancers [4,7,8,14,15,16,17,18,19,20]. In preclinical and clinical trials, it has demonstrated that the inhibitors of c-MET have antitumor activity in treatment of NSCLC. And for EGFR-TKIs (epidermal growth factor receptor-tyrosine kinase inhibitors) resistant and EGFR-TKI naive NSCLC patients, combination use of c-MET inhibitors and EGFR-TKIs (EGFR inhibitors) may be considered as a promising treatment option [20]. In the past years, many c-MET inhibitors have being discovered and developed to suppress tumor [21,22,23,24,25]. For example, Balan et al. found that Honokiol inhibited calcineurin inhibitor-induced renal tumor growth by suppressing c-MET-Ras-HO-1 axis [23]. Therefore, finding effective c-MET inhibitors and understanding the regulation of HGF/c-MET pathway are crucial for further inhibiting prolonged activation of this pathway in human cancers, including digestive system cancers.

In 1993, first microRNA (miRNA) was discovered and identified, then functional studies of miRNA has been expanded in recent years [26,27]. Importantly, the roles of miRNAs in different tumor progression have been reported [28]. Several publications have reported that several miRNAs inhibit digestive system cancer progression through targeting c-MET [29,30,31,32]. For example, Korhan et al. revealed that restoration of miR-181a-5p significantly suppressed hepatocellular carcinoma via directly targeting c-MET [31]. In the future, the miRNAs-HGF/c-MET axis could serve as a potential target for digestive system cancer treatment.

In the current review, the physiological and pathological mechanisms of the aberrant HGF/c-MET in digestive system cancers have been described. Specifically, the new therapies using c-MET inhibitors have been highlighted and some special miRNAs that mediate c-MET in partial digestive system cancers have been summarized.

## 2. HGF/c-MET Inhibitors in Digestive System Cancers

Multiple inhibitors of HGF/c-MET signaling have shown antitumor activity in the preclinical phase of digestive system cancers [33,34]. However, the clinical efficacy of some inhibitors is limited, and some novel HGF/c-MET signaling inhibitors or medication strategies are being developed for digestive system cancers. Clinically relevant examples and some new potential drugs in preclinical trials are listed in Table 1.

## 3. Inhibitors of c-MET/HGF Signaling and Hepatocellular Carcinoma (HCC)

HCC is a significant leading cause of cancer-related death in the world [35]. The therapeutic efficacy of conventional chemotherapy is limited, and five-year survival for advanced HCC is <10% [36]. Sorafenib, a multi-kinase inhibitor (Raf serine/threonine kinases and receptor tyrosine kinases), is the only established standard treatment for advanced HCC [37]; the survival benefit from sorafenib is limited and novel effective therapeutic options need to be developed [36].

Tyrosine kinase receptor c-MET, as a mitogenic growth factor for hepatocytes, has been studied HGF/c-MET signaling increases hepatocyte proliferation and tissue remodeling [35,36]. In addition, *c-MET* is overexpressed at transcriptional and protein levels in HCC [36,38,39]. c-MET inhibitors and multi-kinase inhibitors have been studied for treatment of HCC.

Tepotinib MSC2156119J, highly selective ATP-competitive c-MET inhibitor, has been reported to suppress c-MET-positive HCC tumor in vivo [40]. In Bladt et al.’s research, antitumor effects of MSC2156119J and sorafenib in HCC were compared and evaluated [33]. On the one hand, MSC2156119J effectively inhibits c-MET-positive HCC tumor growth, and also diminished the number of metastatic foci of lung. On the other hand, MSC2156119J monotherapy had superior efficacy compared with sorafenib monotherapy, and the antitumor activity of the combination of MSC2156119J and sorafenib was not superior to MSC2156119J monotherapy In addition, MSC2156119J was also an effective inhibitor for HBV (hepatitis B virus) [33]. Previous reports show that upregulation of HGF is responsible for HBV-induced HCC progression. MSC2156119J is capable of suppressing the HBV-induced HCC possibly through targeting HGF/c-MET signaling [41]. The efficacy of nonclinical studies showed that tepotinib was a promising therapeutic drug in c-MET-positive HCC tumor. In phase Ib trials, tepotinib shows antitumor activity in Asian patients with advanced HCC, including patients with c-MET-positive tumors [42]. Currently, the single-arm phase II trials of tepotinib are continuing in Caucasian HCC patients who are c-MET-positive and sorafenib-insensitive [43]. Overall, tepotinib MSC2156119J shows antitumor activity in nonclinical studies and phase Ib trials in c-MET-positive HCC patients. These results provide evidence for developing a promising antitumor drug for HCC patients.

Tivantinib ARQ197, a non-ATP competitive selective small-molecular inhibitor, not only targets c-MET, but also targets cyclin B1, the proteasome, and glycogen synthase kinase 3 in cancer [44]. In phase I trials, tivantinib shows antitumor activity via suppressing c-MET signaling, promoting apoptosis and inhibiting angiogenesis. Importantly, the safety and tolerability of this drug is qualified. However, the hematological toxicity (mainly neutropenia) of tivantinib was observed in phase II [45]. In Santoro et al.’s phase II trials, 97 patients with advanced HCC and Child-Pugh A cirrhosis were randomly assigned in tivantinib group (360 mg twice-daily or 240 mg twice-daily, capsule) and placebo group in a 2:1 ratio [46]. The median time to progression was longer in the tivantinib group than in the placebo group. For patients with c-MET-positive, the efficacy is more obvious. Inadequately, 10 patients had neutropenia and eight patients had anemia in the tivantinib group, while no patients had neutropenia or anemia in the placebo group. The most common adverse events were more frequent in patients with higher dose tivantinib, and four patients died of severe neutropenia [46]. The antitumor efficacy of tivantinib is promising, however, the dose should be carefully tested and examined and adverse events should be reduced. Recently, in randomized, double-blind and placebo-controlled phase III study, Rimassa et al. reported that tivantinib (120 mg twice daily, tablet) did not improve overall survival in patients with MET-positive advanced HCC [47]. The negative trial might be attributed to tivantinib formulation and dose or adverse events of tivantinib.

SU11274, another small molecular c-MET inhibitor, may inhibit HCC cell growth via suppressing c-MET activation [48]. However, the study about it is only in vitro, and the data of animal models and clinical studies are necessary to be further studied. C-MET inhibitor combined with sorafenib is a potential strategy for HCC treatment. A novel c-MET inhibitor DE605 plus sorafenib may effectively induce HCC cell apoptosis in vitro and suppress HCC tumor xenografts in vivo. On the one hand, DE605 in combination with sorafenib improves therapeutic efficacy (inhibiting proliferation and inducing apoptosis) for HCC. On the other hand, sorafenib inhibits DE605-mediated ERK (extracellular regulated protein kinases) activation [49]. C-MET inhibitor DE605 combined with VEGF inhibitor sorafenib may enhance efficacy and reduce side effects. Therefore, drug combination may be a better strategy for HCC treatment.

Medicinal peptide LZ8, extracted from the Chinese herbal drug *Ganoderma lucidum*, has antitumor activity in breast cancer, lung cancer, cervical cancer, and HCC [50,51,52,53]. Unlike other c-MET inhibitors, LZ8 prevents the tumor progression in HCC without c-MET dependent [53]. As a Chinese herbal ingredient, the safety of the drug has been certified, however, it is still required for clinical trials to verify safety and efficacy in HCC patients.

Other c-MET inhibitors, such as cabozantinib capmatinib, golvatinib, and foretinib, also have been reported for HCC treatment [54,55,56,57,58]. All of these c-MET inhibitors show antitumor activity in nonclinical studies in HCC. However, the efficacy and safety of these drugs is unpredictable. Firstly, side effects (liver and bone marrow toxicity, neutropenia, and anemia) caused by c-MET inhibitors have been frequently reported [53]. Secondly, HCC is a heterogeneous disease, individual c-MET-based target therapy has limited benefit for HCC patients, especially for patients with c-MET-negative. Finally, advanced HCC usually is accompanied with fibrosis and cirrhosis, which reduces phase I and phase II metabolic enzyme activity and hepatic drugs clearance [59].

## 4. Inhibitors of c-MET/HGF Signaling and Gastric Cancer

In gastric cancer, c-MET expression elevation also is a poor overall survival marker, comparing with c-MET-negative tumors [60,61]. Several c-MET inhibitors have been developed as anticancer drugs in gastric cancer treatment.

Tivantinib ARQ197 has been investigated in several clinical trials in different cancers, including NSCLC, HCC and metastatic gastric cancer. In an open-label, multicenter trial phase II, 31 Japanese and Korean patients with metastatic gastric cancer were enrolled; 11 patients achieved disease control [62]. However, the adverse events were still serious.

A Phase I trial of another c-MET inhibitor SAR125844 enrolled 22 Asian patients with gastric cancer; it showed modest antitumor function in two patients with MET-positive gastric cancer, adverse events were common in these patients [63]. Similar to the clinical situation of HCC treatment, most of the c-MET inhibitors have limited benefit for treatment of cancer because of adverse events and c-MET-positive limitation. More and more c-MET inhibitors have been found to have antitumor activity in gastric cancer, but many drug studies have only stayed in nonclinical stage.

Gavine et al. reported that 6% of *c-MET* genes were amplified and 13% of proteins were overexpressed in Chinese gastric cancer patient tumors [64]. The highly selective c-MET small molecule inhibitor volitinib is capable of inhibiting proliferative activity of gastric cancer cells via avoiding phosphorylation of c-MET, and thus inhibiting the activation of c-MET signaling and downstream pathway (AKT and ERK). Volitinib shows antitumor efficacy in patient-derived tumor xenograft models [64].

Toiyama et al. have shown c-MET was predominantly expressed in the transmembrane area, while HGF was mainly expressed in the cytoplasm of primary gastric cancer cells [65]. Importantly, they confirmed overexpression of HGF and c-MET are correlated with peritoneal dissemination and poor prognosis in gastric cancer. The c-MET inhibitor SU11274 may suppress peritoneal mass in gastric cancer nude xenograft model [65]. In parallel, Yashiro et al. studied the efficacy of the c-Met inhibitor SU11274 in combination with irinotecan and found that they have superior efficacy in suppressing in vivo tumor growth by SP-enriched cell lines (OCUM-2M/SP cells) than either group monotherapy [66]. In terms of mechanisms, SU11274 inhibited c-MET signaling and thus decreased the expression of uridine 50-diphosphate-glucuronosyltransferase 1A1 (UGT1A1), which was related with drug resistance to irinotecan [66]. Desirably, the clinical effects of SU11274 monotherapy and combined SU11274 with irinotecan need to be further explored.

Moreover, other selective small molecule c-Met inhibitors such as KRC-408, KRC-00715, and Simm530, and multitargeted kinase inhibitor T-1840383, suppressed the growth of gastric cell lines and tumor xenograft [21,67,68,69]. Also, KRC-00715 and Simm530 suppressed specifically the proliferation of c-MET-overexpressed gastric cells [21,67]. Consistently, these c-MET inhibitors suppress c-MET phosphorylation and its constitutive downstream effectors AKT and ERK [21,67,68,69].

## 5. Inhibitors of c-Met/HGF Signaling and Colorectal Cancer (CRC)

CRC is the fourth most common cancer in the world [70]. High expression of c-MET is associated with tumor invasion and lymph node and hepatic metastasis in CRC [71]. Growing data suggest suppression of c-MET activation may inhibit tumor activity for patients with colorectal carcinoma.

It has been mentioned that SU11274 may suppress tumor cell growth in HCC and gastric cancer via specifically inhibiting c-MET phosphorylation, and the antitumor function also been observed in CRC [48,65,66,72,73]. Gao et al. reported that SU11274 showed inhibitory effects on cell proliferation in four colon cancer cell types [73]. In parallel, Gao et al. described SU11274 induced G1-phase arrest and restrained cell survival in vitro, and suppressed the growth of the xenograft tumor in vivo [72]. In these studies, the clinical efficacy and safety of SU11274 is still unclear. So further studies are necessary to explore the effects of SU11274 in colorectal cancer patients.

In addition to SU11274, the clinical effects of tivantinib have been explored in several human cancers, including CRC. In a placebo-controlled and phase 1/2 study, Cathy et al. reported the efficacy of tivantinib combined with irinotecan and cetuximab in patients with *KRAS* (kirsten rat sarcoma viral oncogene) wild-type metastatic colorectal cancer. However, the progression-free survival has not been significantly improved [74]. Tivantinib is probably not suitable for this CRC subgroup.

Commonly, c-MET is co-present with EGFR in 78% to 80% in CRC [70]. Qiu et al. revealed that Norcantharidin (NCTD) suppressed cell proliferation and induced G2/M phase arrest via decreasing the levels of the total EGFR, the activated EGFR, the total c-MET, and the activated c-MET in colon cancer cells (HCT116 and HT29 cells) [70]. As a c-MET inhibitor, NCTD is the demethylated analogue of Cantharidin and has stronger antitumor activity and much less urological toxicity than Cantharidin [70,75]. Therefore, NCTD may be a promising therapeutic drug and its clinical effect and safety is expected.

Mounting evidence supports the notion that c-MET inhibitors play significant roles in human cancers, including CRC. The reports of HGF inhibitors are relatively rare. Owusu et al. demonstrated that an inhibitor of HGF (SRI 31215) inhibited fibroblast-induced MET activation and DU145 cell EMT and migration. Meaningfully, SRI 31215 overcomes autocrine HGF/MET signaling-mediated the primary resistance to EGFR inhibitors (cetuximab and panitumumab) in colon cancer cells [76]. SRI 31215 might be a useful drug candidate to colorectal cancer patients, in combination with EGFR inhibitors, may improve drug efficiency and overcome drug resistance.

Overall, although the studies of HGF/c-MET inhibitors are still in the preclinical stage, they may serve as a potential direction for CRC treatment.

## 6. Inhibitors of c-Met/HGF Signaling and Pancreatic Cancer

Pancreatic ductal adenocarcinoma (PDAC) is one of the most lethal malignancies in pancreatic cancer [77]. The frequency of c-MET overexpression in PDAC is generally high (80%), and c-MET levels have been considered as an indicator for overall survival and recurrence rates for PDAC patients [78]. Hage et al. reported that c-MET inhibitor cabozantinib increases gemcitabine efficacy and gemcitabine resistance in pancreatic cancer [77]. On the one hand, combination of gemcitabine and cabozantinib exhibits more pronounced antitumor efficacy than monotherapy. On the other hand, cabozantinib overcomes gemcitabine resistance of pancreatic cancer cells by inducing apoptosis and suppressing total c-MET, the phosphorylated c-MET and reprogramming transcription factor *SOX2* [77].

Crizotinib, a c-MET/ALK inhibitor, shows antitumor activity in pancreatic cancer cells via suppressing growth and inducing apoptosis. However, Yan et al. considered that antitumor activity of crizotinib was attributed to targeting ALK signaling not c-MET in pancreatic cancer [78]. In parallel, Avan et al. testified synergistic efficacy of crizotinib–gemcitabine and found that PDAC-3-FM-GC tumor growth was significantly inhibited in the combined drugs-treated mouse group compared with that in treated with crizotinib alone group and gemcitabine alone group [79]. Cytidine deaminase activity contributes to inactivation of gemcitabine, and would reduce the therapeutic efficacy. Notably, crizotinib elevated gemcitabine levels in plasma and tissue specimens via inhibiting cytidine deaminase activity [79]. Synergistic combination of crizotinib and gemcitabine may be an attractive therapeutic direction for PDAC but clinical evaluation is necessary for it to be further investigated. In addition, synergistic combination of tivantinib and gemcitabine also show antitumor activity in cells highly expressing c-MET in pancreatic cancer [80].

Further studies will be needed to verify the pharmacodynamics and safety in clinical trials. And synergistic combination of drugs with c-MET inhibitors may be promising therapeutic strategies in treating human cancers, including PDAC.

## 7. Monoclonal Antibodies against HGF/c-MET in Digestive System Cancers

Several anti-HGF and anti-c-MET monoclonal antibodies direct against extracellular combination of c-MET and HGF have been developed for the inhibition of c-MET-mediated digestive system tumor. Rilotumumab (AMG 102) is a humanized IgG2 monoclonal antibody that selectively binds to HGF [81]. A randomized, placebo-controlled, double-blind phase II clinical trial for patients with advanced gastric or oesophagogastric junction cancer, revealed that rilotumumab plus ECX (epirubicin, cisplatin, and capecitabine) has greater antitumor activity than placebo plus ECX. However, the adverse events were more common in rilotumumab group than that in placebo group [82]. In Catenacci et al.’s investigation, a findings in the clinical phase 3 study (RILOMET-1) showed rilotumumab plus ECX has no effect on improving overall survival for advanced MET-positive gastric or gastro-esophageal junction cancer, and the adverse event frequency was flat with the placebo group [83]. Based on the above research, anti-HGF antibody rilotumumab is not effective on treating of advanced MET-positive gastric cancer.

Onartuzumab is a humanized monoclonal antibody that binds to the c-MET extracellular domain. And onartuzumab has been evaluated in phase III trials in HER2-negative, MET-positive gastroesophageal adenocarcinoma. The combination of first-line mFOLFOX6 (fluorouracil, leucovorin, and oxaliplatin) did not reveal a noteworthy improvement in progression-free survival and overall survival [84]. Similarly, in a randomized double-blind phase II study, Bendell et al. reported that onartuzumab plus mFOLFOX6 and bevacizumab did not significantly improve clinical outcome in metastatic colorectal cancer populations [85].

Furthermore, preclinical studies suggest that anti-c-Met monoclonal antibodies ABT-700 and LY2875358 show antitumor efficacy in solid tumors, including gastric cancer. Both ABT-700 and LY2875358 show preclinical activities via blocking HGF-dependent and HGF-independent MET activation [86,87]. Wang et al. conveyed that ABT-700 showed greater effect on SNU620 cell model in vivo and in vitro than LY2875358 dose [87]. The pharmacodynamics and safety would need to be further validated by clinical trials in the future.

As a consequence, developing anti-HGF and anti-c-MET monoclonal antibodies is still an arduous and demanding task for treatment of digestive cancers.

## 8. Targeting HGF/c-MET Pathway in Digestive System Cancers by MicroRNAs

**Figure 2:**
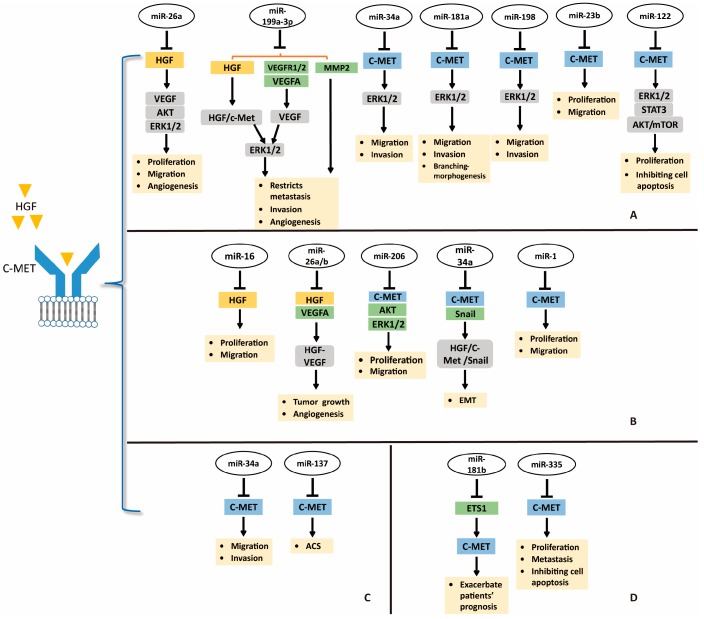
Targeting HGF/c-MET pathway in digestive system cancers by microRNAs. (**A**) targeting HGF/c-MET in HCC by miRNAs; (**B**) miRNAs-HGF/c-MET axis in gastric cancer; (**C**) miRNAs-HGF/c-MET axis in CRC; (**D**) miRNAs-HGF/c-MET axis and pancreatic cancer. Abbreviations: EMT, epithelial–mesenchymal transition; ACS, adenoma–carcinoma sequence. 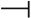 directly target; 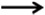 induction.

Despite advances and improvement in techniques to antagonize HGF/c-MET pathway using small molecule inhibitors, many cancers are unresponsive to these inhibitors due to resistance in clinical research [45]. Therefore, novel approaches are required for treatment of cancers. Developing miRNAs as therapeutic targets is a novel and potential direction for suppression of HGF/c-MET pathway in treatment of digestive system cancers; the miRNAs-HGF/c-MET axis is shown in Figure 2.

## 9. Targeting HGF/c-MET in Hepatocellular Carcinoma (HCC) by miRNAs

The miRNA dysregulation is causal in many human cancers including HCC [81]. Because of the strong association between HGF/c-MET activation and human cancers, targeting HGF/c-MET by miRNAs may be a promising approach for cancer treatment (Figure 2) [30]. Yang et al. found that miR-26a could directly target *HGF* to suppress cell viability, migration, and tumor angiogenesis in HCC [88]. Moreover, *miR-26a* inhibits the production of VEGFA in HCC through antagonizing the HGF-c-MET pathway, and the downstream PI3K/Akt/mTOR, ERK, and VEGFR2 signaling. In this sense, miR-26a may well alleviate tumor angiogenesis of HCC via blocking HGF/c-MET pathway and it might be a novel therapeutic strategy for HCC patients with aberrant HGF and c-MET [88]. Similarly, *miR-199a-3p* shows antitumor activity by directly targeting *HGF*, *VEGFR1*, *VEGFA*, *VEGFR2*, and *MMP2* in HCC [89]. Ghosh et al. revealed that *miR-199a-3p* could attenuate HGF/c-MET signaling and thus reduce migration and invasion ability of HCC cells [89]. These reports suggest that targeting miRNAs-HGF axis may be a therapeutic potential for treating HCC.

In parallel, several miRNAs may be tumor-suppressors in HCC via targeting tyrosine kinase receptor c-MET (Figure 2). For example, *miR-34a*, *miR-181b*, and *miR-198* may decrease migration and invasion of HCC cells by directly targeting *c-MET* [32,90,91]. Moreover, *miR-23b* may well suppress HCC cell viability and migration by mediating *c-MET* and urokinase downmodulation [92]. Yang et al. revealed that *miR-122* could decrease cell proliferation and promote apoptosis of HCC cells via targeting *c-MET* and inhibiting its downstream ERK1/2, STAT3, and Akt/mTOR signaling [93]. Conversely, *miR-93* is overexpressed in HCC tumors, and miR-93 level is correlated with c-Met intensity. Ohta et al. verified that *miR-93* may bind to the 3’-UTR of *PTEN* and *Cyclin Dependent Kinase Inhibitor 1A* (*CDKN1A*) mRNA and active HGF/c-MET pathway. Meaningfully, sensitivity of HCC cells to sorafenib and tivantinib was attenuated by elevated *miR-93* [94]. *miR-93*, an oncogenic miRNA, may be a novel therapeutic target for HCC. And combination of sorafenib and tivantinib with *miR-93* inhibitors or anti-miR-93 drugs might improve drug sensitivity and efficacy of the former. All of these reports reveal the important role of miRNAs-c-MET axis in HCC treatment.

## 10. miRNAs-HGF/c-MET Axis in Gastric Cancer

miRNA-mediated suppression of oncogene mRNA translation is one of reasons which make miRNAs as interesting candidates in therapeutics. In gastric cancer, both HGF and c-MET contribute and participate in oncogenic pathways [60,61]. Si et al. reported that *miR-26a* and *miR-26b* may bind to the 3’-UTR of *HGF* mRNA, and thus suppress gastric cancer cell viability and migration in vitro and repressed tumor growth and angiogenesis in vivo through inhibiting HGF-VEGF pathway (Figure 2) [95]. In addition, Li et al. identified that *miR-16* inhibited gastric cancer cell growth and migration by directly targeting 3’-UTR of *HGF* mRNA (Figure 2) [96]. Therefore, miRNAs-HGF axis could function as a novel treatment strategy for gastric cancer.

miR-206 levels were decreased in gastric cancer specimens, however, *c-MET* was overexpressed. Furthermore, the expression of *miR-206* correlates inversely with the expression of c-MET in human gastric tumors. In the study of Zheng et al., *miR-206* may inhibit cellular proliferation, migration, and invasion, and c-MET, CDK4, p-Akt, p-ERK, and p-Rb were inhibited (Figure 2) [29]. In another report, *miR-34a* could inhibit EMT in gastric cancer by targeting *c-MET* and *Snail* (Figure 2). When *miR-34a* was downregulated, HGF/c-MET was activated to induce Snail expression. Additionally, *miR-34a* may directly target *Snail*. These processes blocked E-cadherin expression and contributed to EMT in gastric cancer [97]. Likewise, *miR-1* could also suppress gastric cancer cell growth and migration by targeting MET (Figure 2) [98]. *miR-206*, *miR-34a* and *miR-1* could suppress gastric cancer by mediating *c-MET*, the miRNAs-MET pathway could serve as a promising treatment modality in gastric cancer.

## 11. miRNAs-HGF/c-MET Axis in CRC

The study about the miRNAs-HGF/c-MET axis is in the initial stage, but it has potential to serve as a new direction for treating CRC. Low *miRNA-137* level was confirmed to be correlated with CRC progression by RNA sequencing analysis of 18 colorectal adenoma–carcinoma sequence (ACS) issues. In vitro, *miR-137* is capable of inhibiting CRC cell growth, invasion, and colony formation. In an immunodeficient mouse model, *miR-137* showed antitumor activity, including inhibiting colorectal tumor progression and hepatic metastasis. *c-MET* expression is negatively regulated by *miR-137*, and CRC progression may be suppressed via miR-137-c-MET axis (Figure 2) [99]. *miR-137* also can be silenced by *Mecp-2* (a DNA methyl-CpG-binding protein)-mediated epigenetic function, contributing to colorectal adenoma–carcinoma sequence and tumor progression. *miR-34a* has been described as a tumor-suppressor gene in HCC and gastric cancer by targeting *c-MET*. Consistently, *miR-34a* could negatively regulate invasion and migration of CRC cells via repressing *c-MET* (Figure 2) and long intergenic noncoding RNA (*GAPLINC*) suppresses the function of miR-34a in colorectal cancer [100]. Synthetically, these results provide strong evidence to show the inhibition role of *miR-137* and *miR-34a* in tumor cell migration and invasion in CRC through regulation of *c-MET*.

## 12. miRNAs-HGF/c-MET Axis and Pancreatic Cancer

Pancreatic ductal adenocarcinoma ranks among the most lethal cancers, and its incidence is growing [101]. At present, the relationship between pancreatic cancer and miRNAs-HGF/c-MET is being explored. For example, Cao et al. found that *miRNA-335-5p* could inhibit pancreatic cancer via mediating *c-MET* (Figure 2) [102]. In detail, miRNA-335-5p may directly target the 3’-UTR of *c-MET*, and thus inhibit cell growth, migration, and invasion, and promote apoptosis via activating the HGF/c-MET signaling pathway. Moreover, *NEAT1*, a nuclear-restricted long noncoding RNA, suppresses the microRNA-335-5p/c-MET axis to promote pancreatic cancer malignancy [102]. Consistently, *miRNA-335-5p* also restrains pancreatic cancer cell tumor growth in an xenograft model [102]. In addition, Tomihara et al. reported c-MET expression was induced by irradiation, while the levels of *miR-181b-5p* were reduced [91]. On one hand, preoperative chemoradiation therapy might upregulate c-MET expression in remnant PDAC tissues, and irradiation actives HGF/c-MET pathway by enhancing c-MET expression in PDAC cells. On the other hand, irradiation exposure may reduce *miR-181b-5p* expression and it directly targets the transcription factor *ETS1*, which binds to the *MET* promoter and thus actives c-MET. Hence, *miR-181b-5p* may indirectly suppress the activation of c-MET, and a c-MET inhibitor may be used during preoperative chemoradiation therapy for PDAC patients (Figure 2) [91]. In summary, miRNAs-HGF/c-MET axis would provide more novel treatments for PDAC in the future.

## 13. miRNAs-HGF/c-MET Axis and Other Cancers

The function of miRNAs-HGF/c-MET axis in several human cancers has also been explored, including NSCLC, renal cancer, breast cancer, bladder cancer, and other cancers [103,104,105,106,107]. For example, miR-199a-3p has been mentioned to play an antitumor role in HCC through targeting *c-MET*, *VEGFA*, *VEGFR1*, *VEGFR2*, and *MMP2*. Consistently, *miR-199a-3p* may inhibit the proliferation of HCC cells via targeting *c-MET* and antagonizing downstream signals STAT3, mTOR, and ERK1/2 [107]. Similarly, *miR-335* has been confirmed to inhibit the migration of cancer cells via binding *c-MET* mRNA in pancreatic and breast cancer [102,106]. These reports indicate that miRNAs-HGF/c-MET axis is worth developing for multiple cancer treatments.

## 14. Prospects

The HGF/c-MET pathway is emerging as a therapeutically relevant target in multiple cancers. The discovery and development of c-MET inhibitors has been gradually increasing, including nonselective and selective inhibitors. c-MET inhibitors were diverse for digestive system cancers, however, safety and efficacy have become the main problems in clinical stage studies. New drug developments for c-MET inhibitors offer the potential for better cancer treatment. Clinical trials of these c-MET inhibitors need to be performed to confirm the efficacy and safety. Moreover, insight into the improved safety and efficacy of clinical cancer therapy based on combined medication will provide a new research direction for treating digestive system cancers. In addition, miRNA biology therapy is still in the preclinical stage. Further investigation of the miRNA-HGF/c-MET may supply effective and promising therapy in human cancers, including digestive system cancers.

## Figures and Tables

**Table 1:** Properties of c-MET inhibitors for digestive system cancer treatment.

Type	Agent	Structure	Target(s) of Inhibitor	Patient Population
ATP-competitive small-molecular inhibitor	Tepotinib (MSC2156119J)	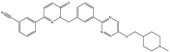	c-MET	Advanced HCC [33]
ATP-competitive small-molecular inhibitor	SU11274	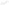	c-MET	Advanced HCC [48] Gastric cancer [65,66] CRC [72,73]
Non-ATP competitive selective small-molecular inhibitor	Tivantinib (ARQ 197)	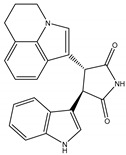	c-MET Gsk 3 Cyclin B1, Proteasome	Cirrhotic patients with HCC [38,45,46,47]
			c-Met (DE605)	Metastatic gastric cancer [62]
				Pancreatic cancer [80]
Combination	Sorafenib and DE605	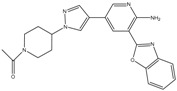 (DE605)		HCC [49]
Medicinal peptide	LZ8	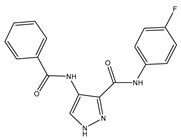	c-MET, ERK, AKT, and JNK	HCC [53]
ATP competitive selective c-Met small molecule inhibitor	Volitinib	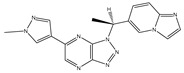	c-MET	Gastric cancer [64]
Selective c-MET small molecule inhibitor	SAR125844	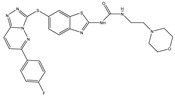	c-MET	MET-amplified gastric cancer [63]
ATP-competitive selective c-Met inhibitor	KRC-408	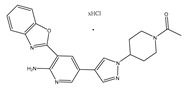	c-MET	Gastric cancer [69]
Selective c-Met inhibitor	KRC-00715	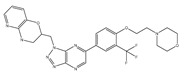	c-MET	Gastric cancer [21]
ATP competitive selective c-Met small molecule inhibitor	Simm530	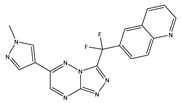	c-MET	Gastric cancer [67]
ATP-competitive multitargeted kinase inhibitor	T-1840383	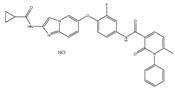	VEGFRs c-MET	Gastric cancer [68]
Synthetic compound	Norcantharidin	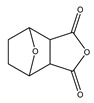	EGFR c-MET	CRC [70]
Triplex inhibitors	SRI 31215	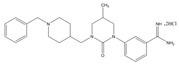	Matriptase, Hepsin, and HGFA	CRC [76]
Combination (ATP-competitive small molecule crizotinib and Gemcitabine)	Crizotinib and Gemcitabine	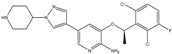 (Crizotinib)	c-MET/ALK	PDAC [78,79]
Nonselective oral multi-kinase inhibitor	Cabozantinib	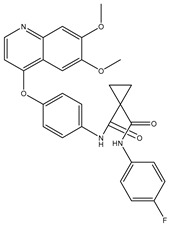	c-MET and VEGFR-2	HCC [58] Pancreatic cancer [77].
